# Classification for long-term survival in oligometastatic patients treated with ablative radiotherapy: A multi-institutional pooled analysis

**DOI:** 10.1371/journal.pone.0195149

**Published:** 2018-04-12

**Authors:** Julian C. Hong, Diandra N. Ayala-Peacock, Jason Lee, A. William Blackstock, Paul Okunieff, Max W. Sung, Ralph R. Weichselbaum, Johnny Kao, James J. Urbanic, Michael T. Milano, Steven J. Chmura, Joseph K. Salama

**Affiliations:** 1 Department of Radiation Oncology, Duke University, Durham, NC, United States of America; 2 Department of Radiation Oncology, Vanderbilt University, Nashville, TN, United States of America; 3 Memorial & St. Elizabeth’s Cancer Treatment Center, Swansea, IL, United States of America; 4 Department of Radiation Oncology, Wake Forest University, Winston-Salem, NC, United States of America; 5 Department of Radiation Oncology, University of Florida, Gainesville, FL, United States of America; 6 Division of Hematology and Medical Oncology, Mount Sinai School of Medicine, New York, NY, United States of America; 7 Department of Radiation and Cellular Oncology, University of Chicago, Chicago, IL, United States of America; 8 Department of Radiation Oncology, Good Samaritan Hospital Medical Center, West Islip, NY, United States of America; 9 Department of Radiation Medicine and Applied Sciences, University of California, San Diego, La Jolla, CA, United States of America; 10 Department of Radiation Oncology, University of Rochester Medical Center, Rochester, NY, United States of America; Ghent University Hospital, BELGIUM

## Abstract

**Background:**

Radiotherapy is increasingly used to treat oligometastatic patients. We sought to identify prognostic criteria in oligometastatic patients undergoing definitive hypofractionated image-guided radiotherapy (HIGRT).

**Methods:**

*Exclusively extracranial* oligometastatic patients treated with HIGRT were pooled. Characteristics including age, sex, primary tumor type, interval to metastatic diagnosis, number of treated metastases and organs, metastatic site, prior systemic therapy for primary tumor treatment, prior definitive metastasis-directed therapy, and systemic therapy for metastasis associated with overall survival (OS), progression-free survival (PFS), and treated metastasis control (TMC) were assessed by the Cox proportional hazards method. Recursive partitioning analysis (RPA) identified prognostic risk strata for OS and PFS based on pretreatment factors.

**Results:**

361 patients were included. Primary tumors included non-small cell lung (17%), colorectal (19%), and breast cancer (16%). Three-year OS was 56%, PFS was 24%, and TMC was 72%. On multivariate analysis, primary tumor, interval to metastases, treated metastases number, and mediastinal/hilar lymph node, liver, or adrenal metastases were associated with OS. Primary tumor site, involved organ number, liver metastasis, and prior primary disease chemotherapy were associated with PFS.

OS RPA identified five classes: class 1: all breast, kidney, or prostate cancer patients (BKP) (3-year OS 75%, 95% CI 66–85%); class 2: patients without BKP with disease-free interval of 75+ months (3-year OS 85%, 95% CI 67–100%); class 3: patients without BKP, shorter disease-free interval, ≤ two metastases, and age < 62 (3-year OS 55%, 95% CI 48–64%); class 4: patients without BKP, shorter disease-free interval, ≥ three metastases, and age < 62 (3-year OS 38%, 95% CI 24–60%); class 5: all others (3-year OS 13%, 95% CI 5–35%). Higher biologically effective dose (BED) (p < 0.01) was associated with OS.

**Conclusions:**

We identified clinical factors defining oligometastatic patients with favorable outcomes, who we hypothesize are most likely to benefit from metastasis-directed therapy.

## Introduction

Metastases remain the leading cause of cancer death worldwide. Metastatic patients are routinely treated with systemic therapy based on the hypothesis that the presence of a single metastasis is universally associated with subclinical micrometastases. However, data and experience suggest that malignant disease burden ranges in spectrum from locoregionally confined disease to widespread distant metastases [1]. Included in this continuum are oligometastatic (OM) patients with metastases limited in number and destination organ who may have a more indolent disease course [2]. Ablative metastasis-directed therapies to all known tumors in OM patients hypothetically could prolong disease-free interval and overall survival. Metastasis-directed therapy of focal OM was first described in surgical series, resulting in long-term disease control and survival for some patients [3,4].

Technological advancements enable the delivery of fewer, more precisely targeted, high radiation doses with steep dose gradients between targets and normal tissues. These radiosurgical-style treatments initially used for brain tumors have expanded to extracranial use. Commonly termed stereotactic body radiotherapy (SBRT) but perhaps more precisely and broadly called hypofractionated image-guided radiotherapy (HIGRT), these treatments are now a standard for many different diseases with high treated-tumor control rates, including non-small cell lung cancer (NSCLC), hepatocellular carcinoma, pancreatic cancer, and prostate cancer [5–9].

With advances in radiation techniques enabling treatment of multiple tumor sites in the same patient [10], HIGRT is increasingly being used to treat all known OM [11]. Prospective studies of OM patients treated with HIGRT report promising rates of treated metastasis control (TMC) and acceptable toxicity rates [12–22]. In these often heavily pretreated populations, similar long-term survival rates as surgical series are seen [23]. However, most patients experience cancer progression [24].

Therefore, optimizing patient selection is critical to define those OM patients most likely to benefit from HIGRT. Although tissue-based biomarkers of the oligometastatic state are beginning to be described, they are not yet ready to be used for patient selection [25–27]. Ongoing randomized trials use only the number of metastases as inclusion criteria. However, given the growing experience treating oligometastases with HIGRT, we hypothesized that other pretreatment (baseline) clinical criteria may exist across different diseases to better define the OM patients most likely to have long-term survival and benefit from metastases-directed therapy. Therefore, we performed a hypothesis-generating analysis of individual patients pooled from multiple institutional experiences, including several prospective trials to identify these criteria.

## Materials and methods

### Patient cohort

Consecutive *exclusively extracranial* OM (≤ 5 metastases) patients treated with definitive-intent radiation to all metastases comprised the study population. Our cohort included patients from multiple prospective clinical trials, including a dose escalation trial [15,28], two prospective pilot studies [14,18], and a phase I/II study of concurrent sunitinib and HIGRT [13,29], as well as patients treated off protocol, but per prior protocols. Patients could receive concurrent non-anthracycline based systemic agents, including sunitinib on protocol. Patients on or off protocol could have had any prior therapy, including definitive metastasis-directed therapy with curative intent or palliative systemic therapy, per the standards of their treating institution. All local institutional review boards (University of Chicago, University of Rochester, Mt. Sinai, Wake Forest University, and the Durham VA) approved this study. Informed consent was obtained for the prospective studies that have been previously published and waived for this pooled analysis.

### Treatment and follow-up

Treatment was per institutional protocols as previously described [13–15,18,28,29]. All patients underwent computed tomography (CT)-based treatment planning in customized immobilization devices with respiratory motion assessment and management where appropriate. The intent of all treatments was to deliver ablative doses to all known metastases. Dose-fractionation schedule varied, with common schedules including ten fractions of 5–6 Gy per fraction, or three fractions ranging from 8–16 Gy per fraction. Patients were followed at standard (approximately 3 month) intervals for toxicity and disease control assessment with physical examination and volumetric imaging including CT and/or PET scanning. Overall survival (OS), progression-free survival (PFS), and treated metastasis control (TMC), on a per patient basis, were defined from the time of HIGRT. PFS was defined as the time to death or any tumor progression, either at a treated metastasis or distant site. TMC was defined as the time to progression at any treated metastasis within a patient. Progression was defined based on the Response Evaluation Criteria in Solid Tumors (RECIST) [30] in the prospective studies [13–15,18,28,29] and based on retrospective review of imaging and clinical documentation for patients who were not treated on trial.

### Statistical analysis

The objective of this retrospective hypothesis-generating pooled analysis was to develop criteria for prognostic risk groups for OS. Statistics were performed in R version 3.3.1 (R Foundation) and source code is included in supplement (S1 File, S2 File) and on GitHub[31]. All statistical tests were 2-sided with significance at p < 0.05. For all OS and PFS analyses, age, sex, primary tumor site, interval to metastasis diagnosis, number of metastases treated, number of treated organs, location of metastasis, prior chemotherapy for primary treatment, prior definitive metastasis-directed therapy, or prior systemic therapy for metastasis were considered. Because data regarding systemic agents were not uniformly collected, this was not included in the analysis. Analysis of TMC did not include age and sex as they were not hypothesized to impact TMC.

OS, PFS, and TMC were assessed with the Kaplan-Meier method, and assessment of variables impacting OS, PFS, and TMC was performed with univariate and multivariate Cox proportional hazards models. Parsimonious multivariate Cox proportional hazards models for each were constructed based on hypothesized clinical relevance, results of univariate analysis, and consideration of stepwise backward regression. The proportional hazards assumption was verified for all individual variables in the final multivariate models by the relationship between Schoenfield residuals. All models additionally globally met the proportional hazards assumption with the exception of PFS. Nonlinearity was assessed with plot of Martingale residuals of the null Cox model for continuous variables in the final OS model (time to metastasis). Patients with missing data were excluded in generating the corresponding univariate and multivariate models.

The binary classification tree approach with recursive partitioning analysis (RPA) was implemented to stratify the patients into risk groups based on OS. The intent of RPA was to identify pre-treatment prognostic classes. Age, sex, primary tumor type, interval to metastasis, number of metastases treated, number of treated organs, location of metastasis, prior chemotherapy for primary treatment, prior definitive metastasis-directed therapy (with either oligoprogressive disease at a treated or new untreated site), or systemic therapy for metastasis were considered as candidates by the RPA calculation, which stratifies based on stepwise binary division of groups based on variables that have more homogeneous outcomes [32]. This is repeated until binary divisions are no longer possible. To minimize overfitting and improve generalizability, the tree was pruned with a cost complexity parameter of 0.018 based on plotting against the cross-validation error. The terminal nodes of the classification tree were selected as the prognostic risk groups. The same procedure was repeated to generate a decision tree based on PFS with a complexity parameter of 0.038. TMC cross-validation error did not reach a local minimum and thus a generalizable model could not be generated. Due to the need for sufficient patients to sufficiently power a hypothesis-generating RPA model, the decision was made to incorporate all patients in the creation of the model rather than using them in a separate validation set.

We also performed an analysis of the relationship between biologically effective dose (BED) and OS, PFS, and TMC, as well as that between TMC and OS and PFS. BED was calculated assuming an alpha-beta ratio of 10 Gy.

$${BED}_{\frac{\alpha }{\beta }}=N\;x\;d\;x\;\left[1+\frac{d}{\left(\frac{\alpha }{\beta }\right)}\right]$$

As different metastases in the same patient could be treated with different doses, the lowest BED per patient was used for this analysis. This was chosen to be as conservative as possible. OS, PFS, and TMC endpoints were analyzed in a univariate fashion based on stratification of BED of 75 Gy or higher (the median BED of the cohort as well as a well-established dose commonly used for patients off protocol[14,18]) with the Kaplan-Meier method and log-rank test. BED and TMC were also included in the final multivariate Cox proportional hazards models for the appropriate endpoints to consider other contributing variables.

## Results

### Outcomes of oligometastatic patients following HIGRT

The multi-institutional cohort included a total of 361 patients. Baseline patient characteristics are presented in **Table 1**. The median follow-up was 26.2 months (35.9 for surviving patients). Primary tumor types included NSCLC (17%), colorectal cancer (19%), and breast cancer (16%). Median time to metastases from initial cancer diagnosis was 12.0 months. Most patients received prior systemic therapy including 74% for primary disease treatment and 70% for metastatic disease. Thirty percent received prior definitive metastasis-directed therapy.

**Table 1:** Baseline demographics and clinical characteristics.

	Total (n = 361)
Variable	Median (Interquartile range)/ Patients (%)
Age (years)	62.7 (54.0–71.0)
Sex	
Male	188 (52%)
Female	173 (48%)
Primary tumor type	
Breast	56 (16%)
Colorectal	69 (19%)
Other gastrointestinal	34 (9%)
Head and neck	34 (9%)
Kidney	25 (7%)
Non-small cell lung cancer	62 (17%)
Prostate	11 (3%)
Sarcoma	22 (6%)
Other*	48 (13%)
Interval to metastatic diagnosis (mos)	12.0 (1.00–36.0)
Number of metastases treated	2 (1–3)
Number of organs treated	1 (1–1)
Metastatic sites (patients may have more than 1)	
Lung	170
Hilum/mediastinum	40
Liver	100
Adrenal	19
Bone	71
Abdominal/pelvic lymph nodes	23
Prior chemotherapy for primary disease	237 (74%**)
Prior definitive metastasis-directed therapy	108 (30%)
Prior systemic therapy for metastatic disease	253 (70%)
BED	75 Gy (65.25–94.5)
Treated on clinical trial	243 (67%)

*Other primary tumor types included: small-cell lung cancer, gynecologic malignancies, carcinoid and neuroendocrine tumors, skin cancer, urinary bladder cancer, adrenocortical carcinoma, malignant peripheral nerve sheath tumor, parathyroid cancer, hemangiopericytoma, thymoma, pituitary malignancy.

**Among those patients with complete information (320)

**Fig 1:**
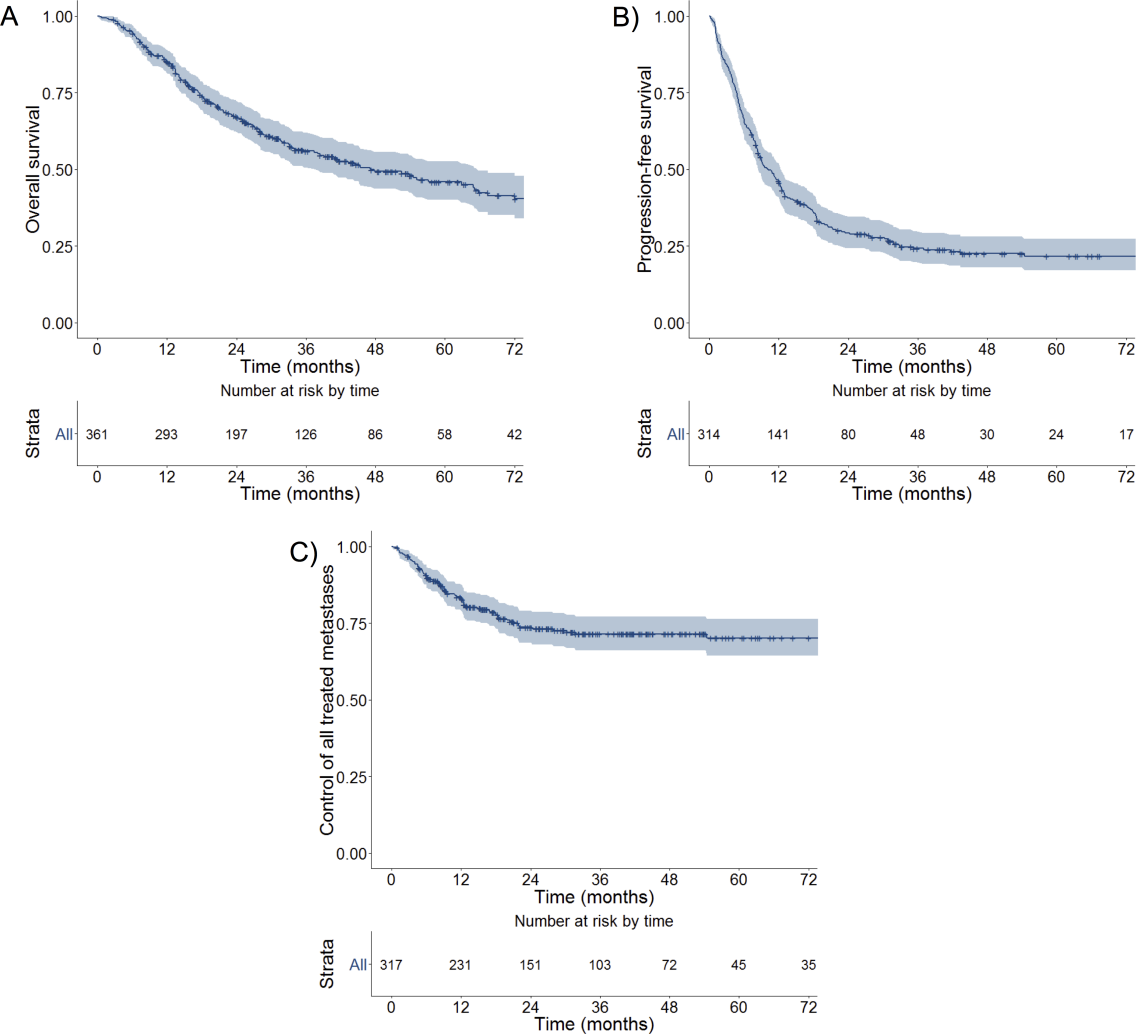
Overall and progression free survival, treated metastasis control for all 361 oligometastatic patients treated with ablative radiotherapy. Median survival was 47.1 months and 3-year survival was 56% (A). Median progression-free survival was 10.1 months and 3-year progression-free survival was 24% (B). Median treated metastasis control was not reached and 3-year TMC was 72% (C).

For the entire cohort, median OS was 47.1 months and 3-year OS was 56% (**Fig 1A**). Median PFS was 10.1 months and 3-year PFS was 24%, which plateaued with a 22% PFS at 5-years (**Fig 1B**). Median TMC was not reached and 3-year TMC was 72% (**Fig 1C**). On univariate analysis, Cox proportional hazards models indicated that compared to breast cancer patients, those with colorectal, other GI, NSCLC, sarcoma, and other primary tumor types had significantly shorter OS (**Table 2**). Other characteristics such as shorter interval to metastatic diagnosis, greater number of treated metastases, greater number of treated organs, hilar or mediastinal lymph node metastasis, and liver metastases were associated with shorter OS. With adjustment on multivariate analysis, primary tumor type, interval to metastatic diagnosis, number of treated metastases, and mediastinal or hilar lymph node, liver, or adrenal metastases were independently significant.

**Table 2:** Univariate and multivariate analysis of overall survival (OS).

	Univariate		Multivariate			
			Pre-treatment		Treatment	
					BED model	
Variable	HR (95% CI)	*p*	HR (95% CI)	*p*	HR (95% CI)	*p*
Age	0.99 (0.99–1.01)	0.69				
Female sex	0.98 (0.73–1.32)	0.89				
Primary tumor type						
Breast	Ref		Ref		Ref	
Colorectal	1.98 (1.12–3.52)	**0.02**	1.83 (0.97–3.44)	0.06	2.18 (1.14–4.14)	**0.02**
Other gastrointestinal	3.49 (1.85–6.58)	**<0.01**	3.80 (1.84–7.86)	**<0.01**	4.30 (2.06–8.96)	**<0.01**
Head and neck	1.77 (0.89–3.52)	0.10	1.81 (0.80–4.10)	0.15	1.93 (0.86–4.37)	0.11
Kidney	0.95 (0.39–2.30)	0.91	1.14 (0.44–3.00)	0.79	1.39 (0.53–3.67)	0.51
Non-small cell lung cancer	2.63 (1.46–4.76)	**<0.01**	2.58 (1.32–5.02)	**<0.01**	3.17 (1.60–6.29)	**<0.01**
Prostate	0.30 (0.04–2.27)	0.24	Insufficient events			
Sarcoma	2.16 (1.07–4.33)	**0.03**	2.66 (1.25–5.64)	**0.01**	2.99 (1.40–6.36)	**<0.01**
Other	2.01 (1.09–3.72)	**0.03**	2.75 (1.40–5.40)	**<0.01**	2.90 (1.49–5.66)	**<0.01**
Interval to metastasis (month)	0.99 (0.98–0.99)	**<0.01**	0.99 (0.98–0.997)	**<0.01**	0.99 (0.98–0.997)	**<0.01**
Number of metastases treated	1.34 (1.19–1.51)	**<0.01**	1.31 (1.12–1.53)	**<0.01**	1.35 (1.16–1.58)	**<0.01**
Number of organs treated	1.60 (1.22–2.10)	**<0.01**				
Any lung metastasis	0.94 (1.07–1.27)	0.68				
Any hilar or mediastinal lymph node metastasis	1.84 (1.18–2.86)	**<0.01**	1.84 (1.06–3.21)	**0.03**	1.49 (0.85–2.61)	0.17
Any liver metastasis	1.45 (1.06–1.99)	**0.02**	1.55 (1.04–2.29)	**0.03**	1.44 (0.97–2.14)	0.07
Any adrenal metastasis	1.70 (0.92–3.13)	0.09	2.27 (1.13–4.54)	**0.02**	1.92 (0.95–3.91)	0.07
Any bone metastasis	0.76 (0.50–1.15)	0.19	1.65 (0.99–2.75)	0.05	1.19 (0.83–2.06)	0.53
Any abdominal or pelvic lymph node metastasis	1.18 (0.60–2.31)	0.64				
Prior chemotherapy for primary disease	1.32 (0.90–1.95)	0.16				
Prior definitive metastasis-directed therapy	0.86 (0.62–1.20)	0.37				
Prior systemic therapy for metastasis	1.23 (0.88–1.71)	0.24				
BED 75 or greater	0.69 (0.44–0.82)	**<0.01**			0.49 (0.33–0.72)	**<0.01**

Abbreviations: BED, biologically effective dose.

PFS data was available for 314 patients. On univariate analysis, primary tumor type was associated with PFS, as was number of treated metastases, number of treated organs, liver metastases, and prior primary disease chemotherapy (**Table 3**). Multivariate analysis demonstrated that primary tumor site, number of involved organs, liver metastasis, and prior primary disease chemotherapy were independent predictors of PFS.

**Table 3:** Univariate and multivariate analysis of progression-free survival (PFS).

	Univariate		Multivariate			
			Pre-treatment		Treatment	
					BED model	
Variable	HR (95% CI)	*p*	HR (95% CI)	*p*	HR (95% CI)	*p*
Age	1.00 (0.99–1.01)	0.49				
Female sex	0.89 (0.69–1.15)	0.37				
Primary tumor type						
Breast	Ref		Ref		Ref	
Colorectal	2.18 (1.38–3.45)	**<0.01**	2.09 (1.32–3.31)	**<0.01**	2.48 (1.53–4.01)	**<0.01**
Other gastrointestinal	2.65 (1.49–4.71)	**<0.01**	2.88 (1.60–5.21)	**<0.01**	3.24 (1.78–5.89)	**<0.01**
Head and neck	2.87 (1.62–5.09)	**<0.01**	3.96 (2.19–7.17)	**<0.01**	4.34 (2.39–7.87)	**<0.01**
Kidney	1.36 (0.73–2.56)	0.34	2.03 (1.04–3.96)	**0.04**	2.38 (1.21–4.71)	**0.01**
Non-small cell lung cancer	2.30 (1.43–3.70)	**<0.01**	2.73 (1.68–4.46)	**<0.01**	3.05 (1.86–5.00)	**<0.01**
Prostate	0.26 (0.04–1.92)	0.19	0.44 (0.06–3.28)	0.42	0.47 (0.06–3.53)	0.46
Sarcoma	3.25 (1.83–5.79)	**<0.01**	4.05 (2.24–7.32)	**<0.01**	4.85 (2.64–8.90)	**<0.01**
Other	2.57 (1.56–4.25)	**<0.01**	3.03 (1.82–5.04)	**<0.01**	3.22 (1.93–5.36)	**<0.01**
Interval to metastasis (month)	0.999 (0.999–1.00)	0.53				
Number of metastases treated	1.18 (1.06–1.31)	**<0.01**				
Number of organs treated	1.47 (1.16–1.86)	**<0.01**	1.42 (1.12–1.80)	**<0.01**	1.36 (1.07–1.72)	**0.01**
Any lung metastasis	1.00 (1.00–0.77)	0.997				
Any hilar or mediastinal lymph node metastasis	1.34 (0.91–1.98)	0.14				
Any liver metastasis	1.39 (1.06–1.82)	**0.02**	1.45 (1.07–1.97)	**0.02**	1.44 (1.06–1.94)	**0.02**
Any adrenal metastasis	1.56 (0.91–2.68)	0.11				
Any bone metastasis	0.77 (0.54–1.11)	0.16				
Any abdominal or pelvic lymph node metastasis	1.35 (0.80–2.27)	0.27				
Prior chemotherapy for primary disease	1.47 (1.08–1.99)	**0.01**	1.57 (1.13–2.19)	**0.01**	1.48 (1.06–2.07)	**0.02**
Prior definitive metastasis-directed therapy	0.93 (0.70–1.24)	0.63				
Prior systemic therapy for metastasis	1.23 (0.92–1.65)	0.16				
BED 75 or greater	0.77 (0.58–1.01)	0.06			0.66 (0.49–0.89)	**0.01**

Abbreviations: BED, biologically effective dose.

TMC data was available and analyzed for 317 patients. Univariate analysis suggested that primary tumor type, number of treated metastases and number of treated organs, liver metastasis, non-bone metastasis, and systemic therapy for metastatic disease were associated with TMC (**Table 4**). With multivariate adjustment, primary tumor type, hilar/mediastinal or liver metastasis, and systemic therapy for metastasis were associated with TMC.

**Table 4:** Univariate and multivariate analysis of per patient treated metastasis control (TMC).

	Univariate		Multivariate			
			Pre-treatment		Treatment	
					BED model	
Variable	HR (95% CI)	*p*	HR (95% CI)	*p*	HR (95% CI)	*p*
Primary tumor type						
Breast	Ref		Ref		Ref	
Colorectal	2.53 (1.22–5.24)	**0.01**	3.03 (1.43–6.43)	**<0.01**	4.71 (2.12–10.46)	**<0.01**
Other gastrointestinal	2.41 (0.95–6.12)	0.06	4.15 (1.57–10.99)	**<0.01**	5.30 (1.98–14.18)	**<0.01**
Head and neck	0.66 (0.18–2.38)	0.52	1.21 (0.33–4.53)	0.77	1.31 (0.35–4.88)	0.68
Kidney	1.60 (0.58–4.41)	0.36	3.22 (1.12–9.24)	**0.03**	5.08 (1.70–15.21)	**<0.01**
Non-small cell lung cancer	1.17 (0.48–2.80)	0.73	1.63 (0.67–3.98)	0.28	2.35 (0.95–5.84)	0.06
Prostate	Insufficient events					
Sarcoma	1.06 (0.33–3.38)	0.92	1.99 (0.60–6.56)	0.07	2.49 (0.76–8.20)	0.13
Other	1.66 (0.72–3.82)	0.24	2.16 (0.93–5.04)	**0.02**	2.64 (1.13–6.19)	**0.02**
Interval to metastasis (month)	0.999 (0.997–1.001)	0.32				
Number of metastases treated	1.21 (1.01–1.45)	**0.04**				
Number of organs treated	1.59 (1.10–2.30)	**0.01**				
Any lung metastasis	0.91 (0.59–1.42)	0.68				
Any hilar or mediastinal lymph node metastasis	1.69 (0.89–3.21)	0.11	2.42 (1.21–4.80)	**0.01**	2.12 (1.06–4.25)	**0.03**
Any liver metastasis	2.47 (1.59–3.85)	**<0.01**	2.05 (1.25–3.36)	**<0.01**	2.15 (1.32–3.49)	**<0.01**
Any adrenal metastasis	0.44 (0.11–1.79)	0.25				
Any bone metastasis	0.43 (0.20–0.94)	**0.03**				
Any abdominal or pelvic lymph node metastasis	1.21 (0.49–3.00)	0.68				
Prior chemotherapy for primary disease	1.72 (0.97–3.07)	0.07				
Prior definitive metastasis-directed therapy	0.61 (0.35–1.06)	0.08	0.61 (0.35–1.08)	0.09	0.57 (0.32–1.01)	0.05
Prior systemic therapy for metastasis	2.38 (1.29–4.41)	**0.01**	2.17 (1.12–4.22)	**0.02**	1.75 (0.89–3.46)	0.10
BED 75 or greater	0.45 (0.29–0.70)	**<0.01**			0.36 (0.22–0.59)	**<0.01**

Abbreviations: BED, biologically effective dose.

### Identifying prognostic cohorts via recursive partitioning

**Fig 2:**
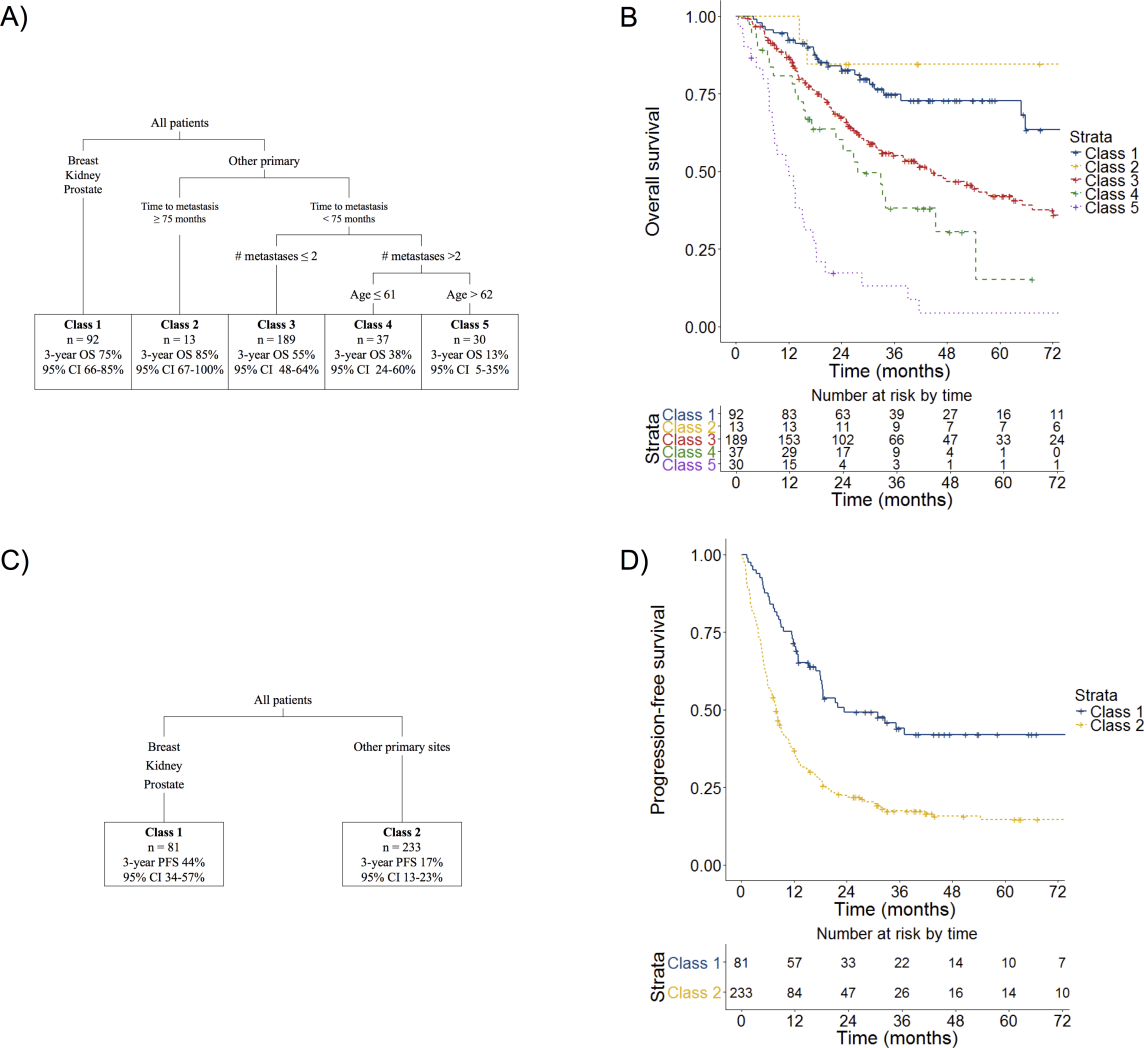
Recursive partitioning models for overall survival and progression-free survival. For overall survival, recursive partitioning allowed stratification of patients into five prognostic classes (A). Overall survival was well-stratified based on RPA class (B); log-rank p < 0.01. For progression-free survival, recursive partitioning allowed stratification of patients into two prognostic classes (C). Progression-free survival was well-stratified based on RPA class (D); log-rank p < 0.01.

Recursive partitioning analysis identified five prognostic classes for overall survival. (**Fig 2A and 2B**). Class 1 (3-year OS 75%, 95% CI 66–85%) consisted of all breast, kidney, or prostate cancer patients (BKP), which RPA separated as distinct from other primary tumor types. Without pruning, RPA suggested that solitary metastasis BKP patients may have superior OS to those with > one metastasis. However, this did not remain following the tree pruning process based on cross-validation error, and thus RPA was unable to further identify prognostic subclasses within class 1. Class 2 (3-year OS 85%, 95% CI 67–100%) included all patients with other diseases but with disease-free interval of ≥ 75 months. Patients with non-BKP disease, shorter disease-free interval (< 75 months), ≤2 metastases comprised class 3 (3-year OS 55%, 95% CI 48–64%). Class 4 (3-year OS 38%, 95% CI 24–60%) included patients with non-BKP disease, shorter disease-free interval, ≥ 3 metastases, and age <62. Finally, class 5 included all remaining patients (3-year OS 13%, 95% CI 5–35%). These differences in overall survival were statistically significant (log-rank p < 0.01). Based on Cox proportional hazards with class 1 as the reference, hazard ratios (HR) were as follows: class 2 0.20 (95% CI 0.04–0.92; p = 0.04), class 3 2.35 (95% CI 1.50–3.67; p < 0.01), class 4 3.51 (1.96–6.29; p < 0.01), and class 5 9.36 (95% CI 5.38–16.27; p < 0.01).

For PFS, RPA defined only two prognostic classes as shown in **Fig 2C and 2D** based on primary tumor type; class 1 (3-year PFS 44%, 95% CI 32–57%): BKP and class 2 (3-year PFS 17%, 95% CI 13–23%): all other non-BKP primary tumor types; log-rank p < 0.01. On Cox proportional hazards, this result was also statistically significant (HR 2.40 with class 1 as reference, 95% CI 1.73–3.34; p < 0.01).

### Impact of BED on survival

**Fig 3:**
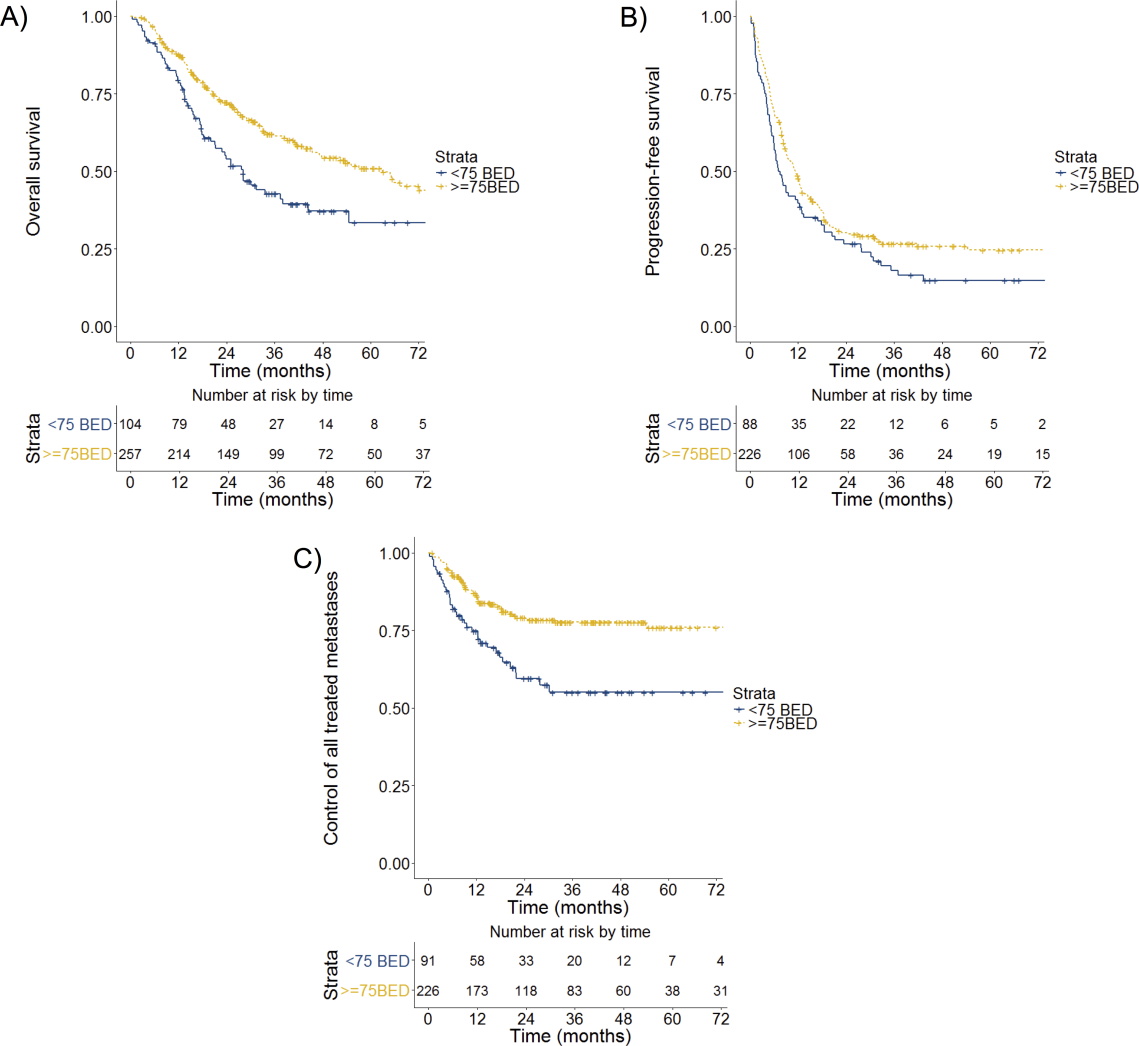
Overall and progression free survival, treated metastasis control by minimum biologically effective dose (BED). BED ≥75 Gy was associated with greater overall survival (p < 0.01) (A) and treated metastasis control (p < 0.01) (C), with trend for progression free survival (p = 0.06) (B).

Univariate stratification by BED suggested a correlation between minimum BED > 75 Gy with OS, PFS, and TMC. Those treated with BED of ≥ 75 had a 3-year OS of 61% (95% CI 55–68%) compared to 43% (95% CI 34–54%) for those treated with BED < 75 (p < 0.01; **Fig 3A**). Three-year PFS for BED ≥75 Gy was 27% (95% CI 21–34%) versus 18% (95% CI 11–29%) for BED < 75 (p = 0.06; **Fig 3B**). With BED ≥75 Gy, 3-year TMC was 78% (95% CI 72–84%), significantly higher than that with BED <75 Gy, 55% (95% CI 44–68%); p < 0.01 (**Fig 3C**). Incorporating BED into the multivariate OS model suggested a statistically significant association (HR 0.49, 95% CI 0.33–0.72; p < 0.01; **Table 2**). This adjusted model including both BED and site of metastasis had a decreased effect size and significance of liver, adrenal, and bone metastases, suggesting potential correlation between higher BED and these treated sites (HR 1.49 (0.85–2.61); p = 0.17). Similarly, the adjusted model for PFS (**Table 3**) suggested a statistically significant association with BED (HR 0.66, 95% CI 0.49–0.89; p = 0.01). In contrast, this appeared independent of other variables in the PFS model.

## Discussion

In this large multi-institutional cohort of *exclusively extracranial* OM patients treated with definitive HIGRT, we found a median progression free survival approaching 1 year and a median overall survival approaching 4 years. Furthermore, 40% of patients were alive *6 years* after metastasis directed therapy with ~20% alive without progression. These data are similar to surgical metastasectomy series [3,4] suggesting a promising role for radiotherapy treating at least limited metastatic patients not technically resectable or medically fit.

We also found specific prognostic factors associated with improved OS, PFS, and TMC. In particular, primary tumor type, time to metastatic diagnosis, number of metastases, age, and metastatic site were independently associated with overall survival. For progression free survival, primary tumor type, number of treated organs, prior chemotherapy for primary disease, and treated liver metastases, were independent prognostic factors. Treated metastasis control was associated with primary tumor type, location of metastasis, and prior systemic therapy for metastasis. Further, we found that higher minimum BED was associated with clinical outcomes.

Our series is unique amongst published reports of prognostic factors for oligometastatic patients. First, we analyzed a large cohort of metastatic patients with a wide range of primary tumors and a variety of *exclusively extracranial* OM sites. This provides a broader characterization of underlying characteristics common to all oligometastatic patients irrespective of primary and secondary tumor sites. Second, the results of the OS RPA demonstrate the importance and interaction of the various pre-treatment prognostic factors identified by Cox proportional hazards modeling. Patients in the most favorable risk group were those with breast, renal cell, and prostate cancer, consistent with findings of a small subcohort of this study [29]. RPA was unable to stratify this cohort further. This suggests that other hypothesized clinical factors such as number of metastases, disease-free interval, age, or metastatic site may not play as large of a role in prognosis for these patients.

These outcomes are favorable in comparison to historical trials, particularly in breast cancer and renal cell carcinoma [24,33,34]. For instance, in a study randomizing patients with metastatic breast cancer to paclitaxel with or without gemcitabine, 91% of patients enrolled had ≤ four metastatic sites with PFS of 8–10 months [35]. While colorectal cancer has been previously suspected to be a favorable disease site and has historically been treated aggressively, RPA did not pool this with BKP diseases. This suggests that patients with colorectal cancer may be less uniformly favorable than BKP diseases and require consideration of additional factors.

For patients with other primary tumor types, a long disease-free interval (≥ 75 months until metastasis development) portended a similarly favorable prognosis. Our cohort of these patients, however, was limited to 14 patients and should be considered hypothesis-generating. For patients with non-BKP primary tumors and shorter disease-free intervals, < three metastases was a favorable prognostic factor. Furthermore, patients with non-BKP primaries, short disease free-intervals, three or more metastases, and age ≥ 62 had nearly a four-fold risk of death in comparison to the entire cohort average, and a nine-fold risk in comparison to patients with BKP diseases. These findings are important as outcomes following metastasis-directed therapy are promising for some, but many patients progress early, stressing the need to better identify patients most likely to benefit.

Beyond the ability to stratify patients, long-term survival in the most favorable populations suggests clinical criteria possibly predicting for a more advantageous biology. The three most favorable classes all had a three-year OS >50%. This indicates that the presented criteria predict for long-term survivors who we hypothesize are the best candidates for aggressive metastasis-directed therapies. However, the 38% 3-year OS of class 4 patients is better than expected for many metastatic patients, indicating that there may be some patients in classes 4 and 5 who could benefit from metastasis-directed therapy to all known metastases.

Prior studies identifying risk groups of OM patients have primarily focused on either specific treated organs or treatment of specific diseases and included intracranial metastases [16,36–42]. Many large series have identified prognostic factors in cohorts of resected pulmonary metastases [3], resected liver metastases [4,16,39], and oligometastatic NSCLC [37,38,43]. Our findings are concordant with and integrate the findings of these studies into a larger framework. Primary tumor type is the key determining factor of our study, with various forms of adenocarcinoma (breast and prostate) portending the best prognosis, consistent with prior data [16]. Lengthy disease-free interval and fewer treated metastases are more detailed indicators of metachronous metastases [16,36–39]. Performance status has been identified in two studies [37,39] as a positive prognostic factor. Although unavailable in our cohort, most patients were treated on protocols requiring high-performance status [13–15,18,28,29]. The consistency of these findings across multiple studies [3,4,16,36–39] and treatment modalities is encouraging. Our findings augment these prior results by demonstrating how the various prognostic factors interact.

High-level evidence supporting ablative therapy for limited metastatic patients is beginning to emerge. A prospective randomized trial recently showed a progression-free survival benefit to consolidative radiation or surgery following systemic therapy for NSCLC patients with three or fewer metastases [44], and thermal ablation of colorectal liver metastases improved survival when given with chemotherapy over chemotherapy alone [45]. Furthermore, data suggest cost-effectiveness of ablative metastasis directed therapy in specific clinical scenarios [46]. However, most patients still experience disease progression. Our data provide a useful and simple tool to aid practitioners in the selection of appropriate candidates for these increasingly implemented treatments [11,47].

This study is limited by available data. Though a large, diverse, and multi-institutional cohort, patients in this cohort were selected for treatment, which might influence the overall results. Moreover, though the diversity of the cohort allows comparisons between a variety of patients and diseases, specific groups are therefore smaller. Application of the prognostic groups, while giving broad guidelines, may not reflect the complete intricacies within each group. For instance, we are unable to capture differences between hormone sensitivity of prostate cancer or biomarker status of breast cancer patients within class 1. Additionally, analyses beyond progression-free survival including freedom from systemic therapy was not available. Finally, the burden of disease in our study was based on number of metastases. It is possible that volume of disease, while correlated, may offer additional value in assessing Nevertheless, our study shows that long-term survivors exist and pre-treatment criteria may facilitate appropriate patient selection. Moreover, though statistical methods were used to attempt to maximize generalizability, our results should be validated on an external independent cohort to verify its applicability to the general population.

Ongoing studies are necessary to assess the benefit of ablative therapy for oligometastases and identify biological factors that may further improve patient selection. Recent data suggest that a microRNA candidate classifier can identify those more likely to survive after HIGRT [25]. Further analyses to expand on the biology of OM patients are ongoing. Additionally, NRG-BR001 (NCT02206334) is currently investigating recommended doses for multiple organ stereotactic ablative radiotherapy, and the randomized phase II SABR-COMET (stereotactic ablative radiotherapy for comprehensive treatment of oligometastatic tumors) has completed accrual (NCT01446744) [48,49]. Finally, given the strong prognostic weight our classifier places on primary tumor type and in particular breast cancer histology, our data validate the need to study ablative metastasis-directed therapy in this population, as is being done in NRG-BR002, randomizing women with 1–2 breast cancer metastases to upfront ablation of all metastases with either surgery or radiation along with standard of care systemic therapy or standard of care systemic therapy alone (NCT02364557) [50].

## Conclusions

In conclusion, in our large multi-institutional cohort, we found that following ablative radiotherapy for oligometastatic patients, long-term survivors exist and a sizable fraction do not progress. We identified prognostic factors for patients undergoing HIGRT for oligometastases. Patients with breast, prostate, or kidney cancers or long disease-free intervals have promising outcomes overall. BED was associated with improved clinical outcomes, and improved treated metastasis control was associated with overall survival.

## Supporting information

S1 File Cox proportional hazards source code. (R)

S2 File Recursive partitioning analysis source code. (R)
